# Aberrant Long-Range Temporal Correlations in Depression Are Attenuated after Psychological Treatment

**DOI:** 10.3389/fnhum.2017.00340

**Published:** 2017-06-28

**Authors:** Matti Gärtner, Mona Irrmischer, Emilia Winnebeck, Maria Fissler, Julia M. Huntenburg, Titus A. Schroeter, Malek Bajbouj, Klaus Linkenkaer-Hansen, Vadim V. Nikulin, Thorsten Barnhofer

**Affiliations:** ^1^Department of Psychiatry and Psychotherapy, Charité—Universitätsmedizin Berlin, Campus Benjamin Franklin Berlin, Germany; ^2^Department of Integrative Neurophysiology, Center for Neurogenomics and Cognitive Research, Vrije Universiteit Amsterdam Amsterdam, Netherlands; ^3^Dahlem Center for Neuroimaging of Emotions, Freie Universität Berlin Berlin, Germany; ^4^Department of Neurology, Max Planck Institute for Human Cognitive and Brain Sciences Leipzig, Germany; ^5^Department of Neurology and Clinical Neurophysiology, Charité—Universitätsmedizin Berlin, Campus Benjamin Franklin Berlin, Germany; ^6^Center for Cognition and Decision Making, National Research University Higher School of Economics Moscow, Russia

**Keywords:** depression, EEG, long-range temporal correlations, theta, mindfulness, stress-reduction

## Abstract

The spontaneous oscillatory activity in the human brain shows long-range temporal correlations (LRTC) that extend over time scales of seconds to minutes. Previous research has demonstrated aberrant LRTC in depressed patients; however, it is unknown whether the neuronal dynamics normalize after psychological treatment. In this study, we recorded EEG during eyes-closed rest in depressed patients (*N* = 71) and healthy controls (*N* = 25), and investigated the temporal dynamics in depressed patients at baseline, and after attending either a brief mindfulness training or a stress reduction training. Compared to the healthy controls, depressed patients showed stronger LRTC in theta oscillations (4–7 Hz) at baseline. Following the psychological interventions both groups of patients demonstrated reduced LRTC in the theta band. The reduction of theta LRTC differed marginally between the groups, and explorative analyses of separate groups revealed noteworthy topographic differences. A positive relationship between the changes in LRTC, and changes in depressive symptoms was observed in the mindfulness group. In summary, our data show that aberrant temporal dynamics of ongoing oscillations in depressive patients are attenuated after treatment, and thus may help uncover the mechanisms with which psychotherapeutic interventions affect the brain.

## Introduction

Spontaneous neuronal oscillations, arising from synchronized activity of large assemblies of neurons are a hallmark of the electrical activity of the brain during rest. Despite their considerable variability and seeming randomness, these oscillations follow characteristic patterns in their temporal structure (Linkenkaer-Hansen et al., 2001). It is now well documented that amplitude fluctuations are correlated over thousands of oscillatory cycles (Nikulin and Brismar, 2005; Palva et al., 2013; Smit et al., 2013), a phenomenon that is being referred to as long-range temporal correlations (LRTC).

It has been speculated that the dynamics of such coordinated activity during rest may serve as an indicator of the adaptability of the neural system, and that a medium degree of LRTC, reflecting a balance of uncorrelated random and strictly correlated patterns, would be optimally adaptive (Linkenkaer-Hansen et al., 2001). Consistent with this view, aberrations in LRTC have been reported in a wide range of psychopathologies, such as Major Depression (Linkenkaer-Hansen et al., 2005), Alzheimer’s disease (Montez et al., 2009), Schizophrenia (Nikulin et al., 2012), and Parkinson’s disease (Hohlefeld et al., 2012). Several studies have compared LRTCs of depressive patients and healthy controls. The majority of these studies report aberrations in LRTC of depressive patients (Linkenkaer-Hansen et al., 2005; Lee et al., 2007; Bornas et al., 2013, 2015; Bachmann et al., 2014), but there are studies that did not find such differences (Hosseinifard et al., 2013). However, research into the nature of these aberrations is still at an early stage with studies differing in methodology and being based on only small samples of patients. For instance, it is currently unclear which are the most relevant frequency bands demonstrating abnormal temporal EEG dynamics in depression, and whether depression is characterized by more persistent (correlated) or more random (decorrelated) temporal dynamics. Thus, further research with larger cohorts seems necessary in order to delineate the nature of aberrations in the temporal structure of spontaneous oscillations in depression. An initial aim of the current study was therefore to compare resting-state assessments of LRTC in currently depressed patients and healthy control participants. Furthermore, given accumulating evidence for LRTC aberrations across a range of mental disorders, it seems important to investigate the possibility to modify them. In a recent study, it was shown that a single session of Neurofeedback training reduces aberrations in alpha LRTC in patients suffering from posttraumatic stress disorder (PTSD; Ros et al., 2016). The results of this study also showed that normalization of alpha LRTCs in PTSD patients was associated with symptom relief.

Given these encouraging findings, the second aim of our study was to investigate the modification of LRTC by means of therapeutic interventions. A particularly promising approach in this context might be training in mindfulness meditation. It is aimed at establishing an open and acceptant awareness of present-moment experience and has been introduced into clinical contexts as a means of helping patients to become better at decentering from engagement in maladaptive repetitive patterns of thinking (Teasdale, 1999). The practice counters habitual tendencies of drifting into mind wandering and, therefore, may have effects that could potentially generalize to the dynamics of neural processes during rest. Indeed, previous research has demonstrated effects on resting-state brain activity in clinical and non-clinical samples (Davidson et al., 2003; Barnhofer et al., 2007). In order to test changeability in the current study, we allocated depressed participants to either participate in a brief mindfulness meditation intervention or an active control condition, in which participants were guided to reduce stress by taking regular periods of rest, and assessed LRTC again after the psychological treatments for depression, both of which were 2 weeks in duration. This allowed exploring the stability of LRTC under conditions of change in severity of symptoms. The current study focused on patients with chronic and recurrent depression following the assumption that changes in resting-state activity of the brain might be particularly pronounced in this group.

## Materials and Methods

### Participants

Depressed participants and healthy control participants were recruited through advertisements in newspapers, on the Internet, and in public transport. Before participants were included in the study they were screened by Structured Clinical Interview for DSM IV (First et al., 2002). Details about inclusion and exclusion criteria and the recruitment procedure are provided in Supplementary Material S1. Clinical and socio-demographic characteristics of the final sample are shown in Table 1.

**Table 1:** Sociodemographic characteristics, course characteristics and current use of antidepressants in depressed participants with valid EEG data who completed the mindfulness training (*n* = 36) and depressed participants with Valid EEG data who completed the stress reduction training (*n* = 29).

Characteristic	Mindfulness training	Stress reduction training	*df*	Test statistic	*p*	Effect size
Age, *M* (*SD*)	41.6 (12.8)	42.3 (11.8)	63	*F* = 0.05	0.82	*η*^2^ = 0.001
Gender, *n*_female_ (*%*)	23 (63)	16 (55)	1	*χ*^2^ = 0.51	0.48	
Age of onset, *M* (*SD*)	17.3 (8.5)	17.2 (11.2)	63	*F* = 0.001	0.97	*η*^2^ = 0.000
Number of previous episodes, Med [range]	6.5 [1, 14]	6 [2, 35]		Median test	0.91	
Current use of antidepressants, *n* (%)	10 (27)	9 (31)	1	*χ*^2^ = 0.08	0.77	
*Tricyclics*	*2*	*1*				
*SSRIs*	*4*	*6*				
*SSNRIs*	*3*	*2*				
*Anticonvulsants*	*1*	*0*				

### Procedure

Depressed and healthy control participants who met the respective inclusion criteria participated in the baseline EEG assessment. Depressed participants were then randomly allocated to one of two treatment conditions, brief mindfulness training or stress-reduction that included psycho-educational interventions. The 2-week interventions were delivered in a series of three 1.5-h weekly individual sessions and included intensive daily home practice. Participants of both groups received a booklet that described in detail the practices for each day along with their rationale and related psycho-educational material.

Participants in the mindfulness training engaged in formal meditation practice for about 25 min twice per day on 6 out of 7 days of each of the 2 weeks using recorded guided meditations. Practices were shorter in duration than the practices in Mindfulness-Based Cognitive Therapy (MBCT; Segal et al., 2002) in order to allow for more flexibility in scheduling the practices, but followed the standard sequence of mindfulness-based interventions ranging from focused attention to open monitoring practices with a particular focus on practices that helped patients recognize and disengage from maladaptive patterns of thinking. Participants allocated to the stress-reduction condition were asked to schedule regular periods during which they took time to rest as a means of deliberately retreating from the activities of the day. Length and frequency of the rest periods mirrored the time demands of the meditation training. Participants received a plausible rationale for the stress-reduction training that linked acute depression to stress and suggested ways of using rest, relaxation, and disengagement from negative thinking as an initial and preliminary step towards recovery from depression. Trained clinical psychologists delivered both treatments. For a more detailed description of the interventions see Fissler et al. (2016). The post-treatment assessment was conducted within 1 week after the end of the intervention and followed the same sequence as the pre-treatment assessment apart from including a shortened clinical interview focusing on presence of mood symptoms during the time of the intervention.

The study protocol for the trial was approved by the ethics committee of the Charité University Medicine Berlin, Campus Mitte (EA4/055/13). All participants gave their written informed consent after being informed about the study, first, in writing and then again in through a detailed individual discussion with a clinician. The ethics board of the Charité approved the study without requiring a clinician to judge whether all patients that participated in the study were able to understand the aims and risks of the study. It was therefore not a requirement for the study to have a clinician to make such judgments, yet in practice both of the researchers who consented participants were trained clinicians and would have excluded participants who were unable to understand the aims and risks of the study. None of the potential participants had to be ruled out for this reason.

### Clinical Measures

#### The Structured Clinical Interview for DSM-IV (SCID-I; First et al., 2002)

The SCID is a well-validated semi-structured interview to determine current and past DSM–IV axis-I diagnoses. Interviews were administered by one of two trained clinical psychologists. The SCID was used to assess diagnostic status at baseline and after the end of the interventions.

#### Beck Depression Inventory-II (BDI-II; Beck et al., 1996)

The BDI-II is a widely used self-report measure, to assess severity of current symptoms and consists of 21 groups of statements, referring to the presence of symptoms of depression over the past 2 weeks. Internal consistency in the current sample was *α* = 0.77 at pre-test and *α* = 0.89 at post-test.

#### Ruminative Response Style Questionnaire (RRSQ; Treynor et al., 2003)

Ruminative tendencies were assessed using the RRSQ. The RRSQ includes 22 items that assess the degree to which individuals respond to depressed mood with thoughts that are self-focused, symptom-focused, and focused on the possible causes and consequences of the mood. Internal consistency of the scale in the current sample was *α* = 0.90 at pre-test and *α* = 0.88 at post-test.

### Experimental Condition

For the EEG assessment, participants were seated in a chair and asked to rest with their eyes being closed for a 10 min recording session. A sound indicated the end of the experimental rest condition. Participants were instructed not to try to reach a specific mental state, but rather let the natural flow of thoughts occur.

### EEG Recording

Continuous EEG was recorded from 32 Ag/AgCl active electrode sensors with integrated noise subtraction circuits (actiCap, Brain Products GmbH, Gilching, Germany), placed according to the 10/10 system with a reference electrode located at FCz. Signals were recorded in the frequency range from 0.016 to 450 Hz, and digitized with a sampling rate of 1000 Hz using a Brain Products BrainAmp MR plus (Brain Products GmbH, Gilching, Germany). Electrode impedance was maintained below 10 KΩ.

### EEG Data Analyses

**Figure 1:**
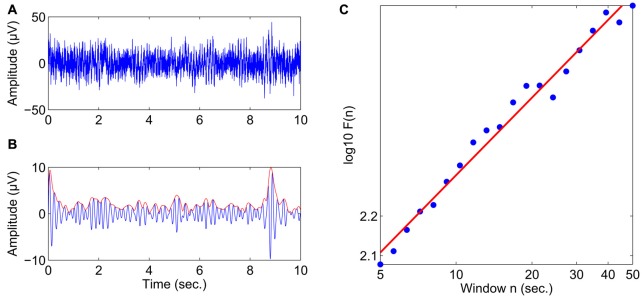
Estimating the scaling exponent of neuronal oscillations with DFA. **(A)** 10 s of broadband EEG from the Fz electrode in a healthy subject. **(B)** The theta-band activity is obtained from the signal in **(A)** using bandpass filtering (4–7 Hz) and the Hilbert transform is used to extract the amplitude envelope (*red line*). **(C)** The DFA fluctuation function, *F*(*n*), scales linearly with window size, *n*, in the range of 5–50 s in double-logarithmic coordinates. The slope of the least-squares line is the scaling exponent, which in this case was 0.65.

Offline analyses were conducted using the Brain Vision Analyzer 2.0 Software (Version 2.0.4.368, Brain Products GmbH, Gilching, Germany) and MATLAB (Version R2010b, The MathWorks, Inc., Natick, MA, USA). Preprocessing of the data consisted of the following steps: The data were downsampled to 200 Hz, highpass filtered at 1 Hz (12 dB/oct) and re-referenced to a common average reference. Vertical and horizontal eye movements were removed from the data using an automated ocular correction approach based on independent component analysis (ICA; Jung et al., 2001; as implemented in the Brain Vision Analyzer software). Next, bandpass filtering and the Hilbert transform were applied to obtain the amplitude envelope of oscillations in the following frequency bands: theta (4–7 Hz), alpha (8–13 Hz), and beta (15–25 Hz) for all 32 electrodes. The temporal structure of these amplitude envelopes was then analyzed using detrended fluctuation analysis (DFA; Peng et al., 1994) as implemented in a custom MATLAB script. DFA estimates the scaling of the root mean-square fluctuation of the integrated and linearly detrended signal across different time windows (for details see Hardstone et al., 2012). Figure 1 provides an illustration of scaling behavior in the amplitude dynamics of oscillatory activity using an example with an almost perfect linear relationship between the logarithm of the time scale and the logarithm of the DFA fluctuation function computed on the amplitude envelope. The slope of the least-squares line in this graph is called the “scaling exponent” (Lux and Marchesi, 1999), which quantifies LRTC. Scaling exponents in the 0.5–1 range indicate a presence of persistent temporal correlations, where larger fluctuations are likely to be followed by larger fluctuations. Uncorrelated signals (e.g., for white noise) have a scaling exponent 0.5. Before DFA, artifactual segments exceeding an amplitude threshold of ±150 μV were flagged as bad, and LRTC were calculated avoiding the inclusion of segments with discontinuous EEG. LRTC were estimated in the range from 5 s to 50 s with 20 windows distributed equidistantly on a logarithmic scale, and control analyses showed that an average of 10.9 (SD 1.2) segments of maximal length (50 s) were maintained per subject and condition after artifact handling.

In addition to the LRTC, we also conducted exploratory analyses of the mean instantaneous amplitude for each frequency band. The purpose of these analyses was to investigate whether the strength of oscillatory activity played a role in depression and to assess to what extent potential effects observed in LRTC were independent from effects observed in time-averaged amplitude modulations.

### Statistical Analyses

In order to compare LRTC and average amplitude in depressed patients and controls, we performed independent *t*-tests for each electrode. A cluster-based permutation approach was applied to account for multiple comparisons (Maris and Oostenveld, 2007). Clusters were defined by the sum of *t*-values of neighboring electrodes (at least one significant neighbor at alpha <0.1). The size of the clusters detected in the actual data was compared to the size of clusters detected in randomly permuted group memberships (1000 random permutations). Separate analyses were conducted for the three frequency bands, and Bonferroni adjusted alpha levels of 0.017 per test were applied to avoid conduction of type I errors.

Pre-post comparisons of treatment-related changes in LRTC and amplitude were investigated using paired *t*-tests for single electrodes with the cluster-based permutation approach described above performed to account for multiple comparisons.

Furthermore, we explored the relations between treatment-related changes depressive symptomatology and LRTC difference scores in the observed clusters using Pearson’s coefficient.

## Results

### Comparisons between Depressed Patients and Healthy Controls

Valid EEG data at baseline were available for 71 of the 74 depressed participants, and for all of the 25 healthy control participants. Socio-demographic and clinical characteristics of all participants are provided in Table 1.

**Figure 2:**
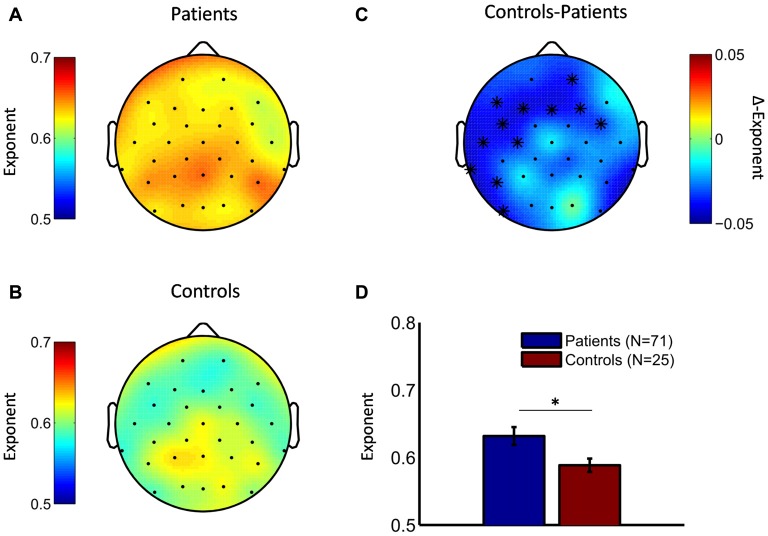
Depressive patients exhibit stronger long-range temporal correlations (LRTC) in theta oscillations compared to healthy controls. Topographic distributions of the DFA exponents point to strongest LRTC in theta oscillations in the parietal region both in depressive patients **(A)** and healthy controls **(B)**. **(C)** The difference topography (controls minus patients) indicates that patients had stronger LRTC in many cortical areas. Cluster-based permutation statistic revealed a significant cluster in left temporal and frontal regions (cluster-electrodes are marked with a star). **(D)** Mean exponent values at a central cluster-electrode (FC5, *t*-statistic, *p < 0.05).

Comparison of the scaling exponents in healthy controls and depressed patients showed elevated LRTC of theta oscillations in the patient group (cluster statistic, *p* = 0.011). No differences were observed for alpha and beta LRTC. The spatial distribution of the scaling exponents for theta oscillations in depressed patients and healthy controls is shown in Figures 2A,B, respectively. In both subject groups, the spatial maximum of the exponent was over the parietal areas. Differences between groups in theta LRTC were observed at multiple sensor locations including mid and left frontal as well as left temporal electrode sites (Figures 2C,D). Exploratory analyses of the time-averaged amplitude of neuronal oscillations showed no differences between the depressed and control groups in any of the examined frequency bands (data not shown).

In order to explore the influence of antidepressant medication on LRTC, we compared scaling exponents in patients who were taking antidepressant medication at the time of the study and those who were not taking medication. There were no significant differences in scaling exponents and mean amplitude in any of the frequency bands between these two groups.

### Pre- to Post-Treatment Changes in Chronically Depressed Patients

Analyses of pre- to post-treatment changes were based on participants who had completed the treatment and had valid EEG data (*N* = 65; *n* = 36 in the mindfulness group and *n* = 29 in the stress-reduction group). The final treatment groups did not differ in socio-demographic and clinical characteristics as shown in Table 1. Importantly, the number of patients that used antidepressant medication during the study did not differ between treatment groups, and numbers indicated no obvious group differences in the different types of antidepressant medication (see Table 1).

Pre- to post-changes of self-reported depression and rumination were analyzed using repeated-measures analysis of variance (ANOVA) with time as within- and treatment as between-subjects factor. Analysis of changes in BDI-II scores yielded a significant main effect of time (*F*_(1,63)_ = 143.5, *p* < 0.001), treatment (*F*_(1,63)_ = 14.1, *p* < 0.001), and a significant time by treatment interaction (*F*_(1,63)_ = 12.6, *p* < 0.001), due to stronger reductions of BDI-II scores in the mindfulness group, than in the stress reduction group (see Table 2). Analysis of the RRSQ rumination scores, revealed a significant main effect of time (*F*_(1,63)_ = 7.8, *p* = 0.007), no main effect of treatment, and a significant time by treatment interaction (*F*_(1,63)_ = 9.1, *p* = 0.004), due to significant decreases in the mindfulness group and no significant change in the stress reduction group (see Table 2). We applied the Lilliefors test to check our psychological variables for normality. At baseline, BDI and RRSQ values were confirmed to come from a normal distribution, the same was observed for post-treatment RRSQ values. Post-treatment BDI values showed a slight deviation from a normal distribution. There were no outliers in any of the datasets. It has been shown that ANOVA is robust against moderate deviations from normality; simulation studies show that false positive rate is not affected considerably by violations of the normality assumption (Harwell et al., 1992).

**Table 2:** Means and standard deviations of depression and rumination scores at pre- and post-assessment in the mindfulness (*n* = 36) and stress reduction groups (*n* = 29).

	Mindfulness		Stress reduction	
	Pre	Post	Pre	Post
BDI-II	27.4 (7.1)	9.8 (6.3)***	28.9 (6.9)	19.4 (9.3)***
RRSQ	57.2 (11.4)	48.8 (11.9)***	56.9 (11.2)	54.1 (14.2) ns.

Note. BDI-II, Beck Depression Inventory II; RRSQ, Ruminative Response Style Questionnaire. ***p < 0.001, ns., not significant.

**Figure 3:**
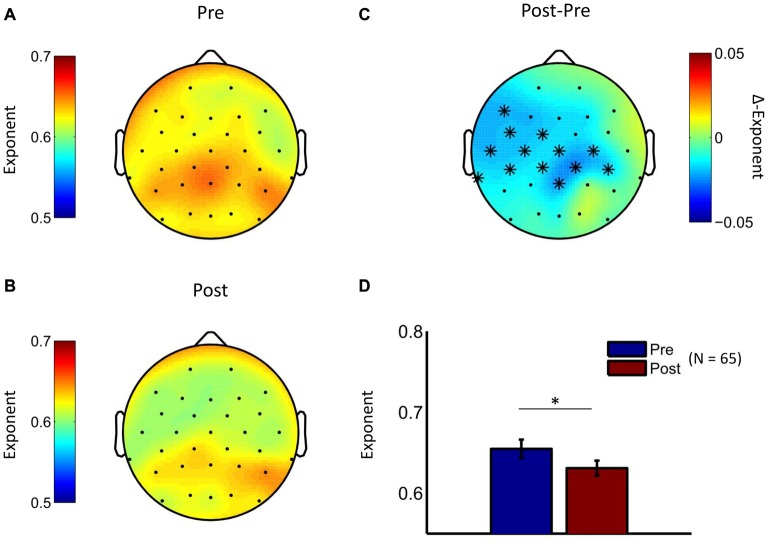
LRTC in theta oscillations are reduced by psychological treatment. The topographic distribution of DFA exponents in theta oscillations is similar for the baseline measurement **(A)** and the post-treatment measurement **(B)** for the pooled treatment groups. **(C)** The difference topography (Post minus Pre) indicates widespread reductions in LRTC due to interventions. Cluster-based permutation statistic revealed a significant cluster at widespread electrode locations (cluster-electrodes are marked with a star). **(D)** Mean exponent values at a central cluster-electrode (Pz, *t*-statistic, *p < 0.05).

Given that both interventions were associated with pronounced reduction in depression scores, we conducted an initial EEG analysis in which we pooled the treatment groups in order to test whether temporal brain dynamics had also changed. Indeed, analysis of pre- to post-treatment changes in scaling exponents showed LRTC reduction in the theta frequency band (cluster statistic, *p* = 0.006). No significant clusters were observed in the other two frequency bands. The cluster in the theta frequency range was observed at centroparietal, left frontal, central, and temporal electrodes (Figure 3), thus suggesting an attenuation of LRTC in depressed patients towards the level of healthy controls reported above. Exploratory analyses of the time-averaged amplitude of neuronal oscillations showed pre- to post-treatment changes in the beta frequency range (see Supplementary Figure S2).

**Figure 4:**
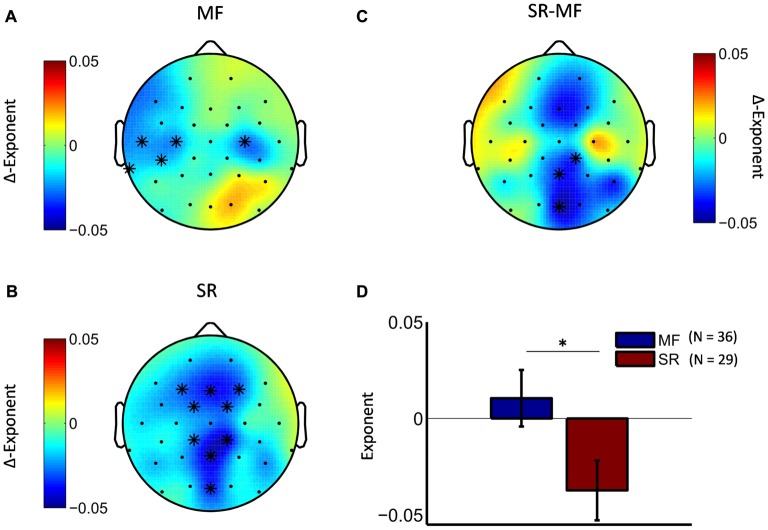
Mindfulness training and stress-reduction differentially affect the topography of LRTC in theta oscillations. The difference topography in the mindfulness group **(A)** and the stress reduction group **(B)** shows distinct topographic patterns (significant pre-post differences are marked with a star). Direct comparison of the two groups revealed a marginally significant cluster **(C)**, which was located at posterior midline electrodes (cluster-electrodes are marked with a star). **(D)** Mean ∆-exponent values at a central cluster-electrode (Oz, *t*-statistic, *p < 0.05).

Next, we tested whether the observed effect in the theta frequency range differed between the two treatment groups. Cluster-statistics, conducted on the pre-post differences scores of the two groups, revealed a marginally significant cluster (cluster statistic, *p* = 0.09) at posterior electrode locations (Figures 4C,D). Explorative analyses of the pre-post differences in separate treatment groups showed that in the stress-reduction group theta LRTC attenuation was mainly observed along the fronto-parietal midline region (Figure 4B), while in the mindfulness group we observed a bilateral attenuation of theta LRTC in central and temporal regions that were more pronounced over the left hemisphere (Figure 4A).

### Correlational Analyses

**Figure 5:**
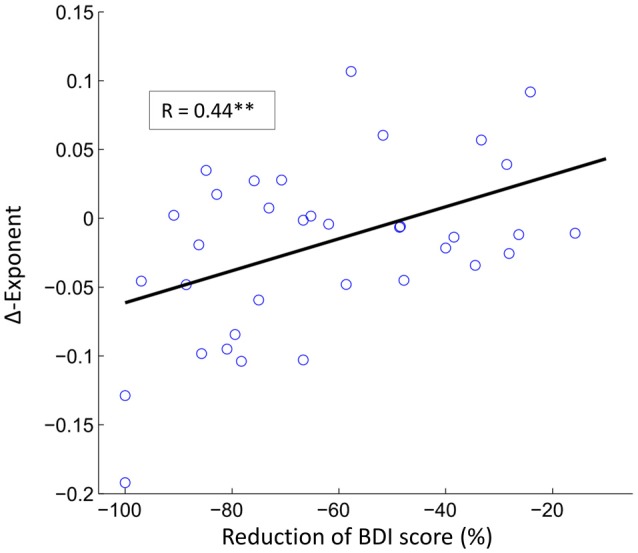
The reduction in depression symptoms correlate with reductions in LRTC in theta oscillations. The scatter plot shows the relationship between reduction of BDI score and the reduction of theta LRTC in the mindfulness treatment group (Pearson correlation coefficient, *r* = 0.44, **p < 0.01) at electrode TP9.

Finally, we investigated whether changes in theta LRTC were associated with the pre- to post-treatment changes in depressive symptomatology (BDI-II) or rumination (RRSQ). These analyses were restricted to the electrodes that showed significant pre to post changes and alpha levels were adjusted using Bonferroni correction. The only significant finding was a positive correlation between BDI-II score reduction and reduction of theta LRTC within the mindfulness group (see Figure 5).

## Discussion

The aim of the present study was to examine differences in LRTC in neuronal oscillations between depressed patients and healthy controls, and to investigate treatment-related changes in LRTC in depressed patients after attending a brief mindfulness or stress reduction training. To our knowledge, this is the largest study so far to investigate differences in LRTC between depressed patients and healthy controls, and the first to investigate effects of psychological interventions on this parameter. In line with previous research, our findings show significant aberrations in the temporal structure of spontaneous oscillatory brain activity in depressed patients as compared to healthy controls, and extend previous research by indicating that such aberrations are likely to disappear as patients recover from the acute state of depression.

Differences in LRTC between depressed patients and healthy control participants indicated more persistent LRTC in patients thus adding to the existing evidence indicating that depression is characterized by changes in the temporal dynamics of resting-state neuronal oscillations (Mumtaz et al., 2015). We surmise that increased LRTC in depression is reflective of a reduced ability to switch transiently between brain states, a deficit that may be related to the increased persistence of maladaptive thinking observed in depression. In contrast, the medium levels of LRTC in healthy subjects may reflect relatively faster switching between neuronal states, which should reduce the likelihood of being drawn for a long time into a given state. Interestingly, our findings in depressed patients contrast with what is observed in patients with schizophrenia (Nikulin et al., 2012), where LRTC were attenuated (compared to healthy subjects), potentially reflecting unusually fast changes in neuronal states, which may indicate tendencies towards disorganized thinking.

Previous studies on LRTC in depression reported both, more persistent (e.g., Lee et al., 2007), and more random (e.g., Linkenkaer-Hansen et al., 2005) temporal dynamics in depressive patients, suggesting reductions in adaptability of the neural system in depression that could be due either to rigidity or lack of associations. The reasons for these conflicting findings are unclear at this point although there are a number of factors with regard to which the studies differed. It is important, for example, to keep in mind that the current study was based on a sample of patients with a chronic or highly recurrent course of the disorder, where brain patterns characteristic of depression may have become more engrained and rigid over time. Patient samples for the other studies were not chosen based on any additional restrictions regarding the course of the disorder. Future research will have to investigate whether aberrations in LRTC are specific to particular aspects or types of depression and what the functional correlates of LRTC aberrations in depression are. With regard to methodological factors, it is noteworthy that the abovementioned studies differ with regard to the length of windows used for the recording of the EEG and the time scales to which the DFA analysis was applied. In the study by Linkenkaer-Hansen et al. (2005), which found reduced LRTC in depression, the duration of the eyes-closed period was 16 min while it was only 10 min in the present study. Longer duration of the eyes-closed state might be associated with more sleepiness and as a result with more alterations in EEG and consequently less pronounced LRTC (Diaz et al., 2013). It is possible, therefore, that LRTC was affected by differences in the psychological states of subjects in the two studies.

In this context it is also worth noting, that the effects were specifically observed in the theta frequency range. The amplitude of theta oscillations has previously been implicated in depression and has been closely linked to working memory and cognitive control in task-based EEG studies (Jensen and Tesche, 2002; Cavanagh and Frank, 2014; Gärtner et al., 2014). It is still unclear whether spontaneous theta fluctuations and their temporal structure during rest are related to such higher order cognitive functions. However, it is an interesting possibility that altered theta LRTC during rest might relate to processes affecting mechanisms of cognitive control in depressive patients.

The results of the pre-to-post treatment analyses showed that aberrations in theta LRTC were reduced after treatment. In healthy controls resting state LRTC has been found to show relatively high temporal stability (Nikulin and Brismar, 2004) and there is preliminary evidence for the heritability of LRTC (Linkenkaer-Hansen et al., 2007). The current data are remarkable from this perspective as they provide an initial evidence that aberrations in LRTC in depressed patients are malleable. The fact that changes occurred after only brief interventions further supports the view that LRTC aberrations in depression predominantly represent a state rather than a trait characteristic. In both of the intervention groups LRTC in the theta frequency range was reduced to levels, which were comparable to those observed in healthy controls, thus indicating complete reversibility. In contrast to our expectations, there was no significant evidence that mindfulness meditation was especially successful in reducing aberrations in LRTC compared to our stress-reduction training that did not include a mental training component. Thus, it is not possible from the current results to clearly attribute the observed LRTC changes to specific treatment effects, as we cannot rule out the influence of third factors. While there was some evidence for a relation between BDI-II score reduction and reduction of theta LRTC, this relation did not emerge in both groups, but was restricted to changes in electrodes that were specifically affected by the mindfulness training, thus indicating a need for further exploration of correlates of change.

In interpreting the current findings, it is important to keep in mind that our study used only minimal interventions. Standard mindfulness trainings consist of 8 weeks of intensive training as compared to the 2 weeks of our intervention, and many of the findings demonstrating effects of meditation training on brain functioning are based on even longer trainings or investigations of expert meditators (Tang et al., 2015). Clinical interventions in acutely depressed patients tend to produce strong effects at the early stages of the interventions, a phenomenon that is referred to as early gains (Hayes et al., 2005), and it is possible that the strong reductions in symptoms observed in our study might have overshadowed more specific effects that resulted from the mental training.

A preliminary indication for differential treatment effects comes from our finding that indicated topographic differences between the two treatment groups. In the stress-reduction group, theta LRTC were mainly attenuated at electrodes close to the midline. The strongest effect was observed at parietal midline electrodes, but the effect was also evident for frontal midline electrodes. In the mindfulness treatment group a different topographic distribution was observed. Theta LRTC reductions in this group were mainly located at left and right central electrodes, and at left temporal electrodes. However, it has to be noted that direct comparison of the two groups only revealed a marginally significant cluster located at posterior midline electrodes, which indicated stronger reduction of theta LRTC in the stress-reduction group in these electrode locations. The relatively small number of electrodes used in this study, the relatively low spatial resolution of EEG recordings in general, and the applied cluster statistic that requires electrodes to be neighbors, might have obscured additional topographic differences between groups. Nevertheless, we think that very different topographic LRTC distribution between the groups should be taken into account for further investigation of treatment effects, considering that the reduction of depressive symptoms and attenuation of the temporal dynamics might depend on the location of the effect. This can be addressed in the future using larger numbers of electrodes in combination with source localization techniques in order to better understand the functional significance of such differences in topographical patterns.

## Conclusion

Altogether, the current results provide further evidence for aberrations of LRTC in depression and add to the current evidence by showing that these aberrations can be modified over relatively brief periods of time. Further research will have to delineate the functional significance of LRTC in psychological terms, and explore effects of mindfulness training on temporal neuronal dynamics using more extensive training programs in order to test whether they might reduce the likelihood of aberrations to recur.

## Ethics Statement

This study was carried out in accordance with the recommendations of the Deutsche Gesellschaft für Psychologie with written informed consent from all subjects. All subjects gave written informed consent in accordance with the Declaration of Helsinki. The protocol was approved by the Charité Ethics Committee.

## Author Contributions

MG supervised the EEG assessment, conducted the data analysis, wrote the manuscript, and approved the final version. MI contributed to data analysis, revised and approved the final version of the manuscript. EW, MF, JMH and TAS conducted the assessments, approved the final version of the manuscript. MB and KL-H revised and approved the final version of the manuscript. VVN supervised the EEG data analysis, wrote the manuscript, and approved the final version. TB supervised the project, wrote the manuscript, and approved the final version.

## Conflict of Interest Statement

The authors declare that the research was conducted in the absence of any commercial or financial relationships that could be construed as a potential conflict of interest.
