# Erratum: Aberrant Long-Range Temporal Correlations in Depression Are Attenuated after Psychological Treatment

**DOI:** 10.3389/fnhum.2017.00509

**Published:** 2017-10-12

**Authors:** Frontiers Production Office

**Affiliations:** Frontiers Media SA, Lausanne, Switzerland

**Keywords:** depression, EEG, long-range temporal correlations, theta, mindfulness, stress-reduction

The publisher failed to include the following affiliation for Vadim V. Nikulin:

**Department of Neurology, Max Planck Institute for Human Cognitive and Brain Sciences, Leipzig, Germany**

The original article has been updated as follows:

Matti Gärtner^1^, Mona Irrmischer^2^, Emilia Winnebeck^3^, Maria Fissler^3^, Julia M. Huntenburg^3^, Titus A. Schroeter^3^, Malek Bajbouj^1^, Klaus Linkenkaer-Hansen^2^, **Vadim V. Nikulin**^4,5,6*†^ and Thorsten Barnhofer^3†^

^1^Department of Psychiatry and Psychotherapy, Charité—Universitätsmedizin Berlin, Campus Benjamin Franklin, Berlin, Germany

^2^Department of Integrative Neurophysiology, Center for Neurogenomics and Cognitive Research, Vrije Universiteit Amsterdam, Amsterdam, Netherlands

^3^Dahlem Center for Neuroimaging of Emotions, Freie Universität Berlin, Berlin, Germany

^4^**Department of Neurology, Max Planck Institute for Human Cognitive and Brain Sciences, Leipzig, Germany**

^5^Department of Neurology and Clinical Neurophysiology, Charité—Universitätsmedizin Berlin, Campus Benjamin Franklin, Berlin, Germany

^6^Center for Cognition and Decision Making, National Research University Higher School of Economics, Moscow, Russia

