# Supplementary material for: Aberrant Long-Range Temporal Correlations in Depression Are Attenuated after Psychological Treatment
Source: Front Hum Neurosci. 2017 Jun 28;11:340. doi: 10.3389/fnhum.2017.00340 (PMC5488389; doi:10.3389/fnhum.2017.00340)
Supplement: Supplementary file 1 [file Data_Sheet_1.docx]

**S1: Recruitment of participants**

Inclusion and exclusion criteria

For depressed participants, inclusion criteria at initial assessment were a) a current diagnosis of Major Depression as assessed by Structured Clinical Interview for DSM IV [[16](#_ENREF_16)], b) a lifetime history of depression with onset before age 19 and either chronic persistence of symptoms or a history of at least three previous episodes of depression, two of which needed to have occurred during the last two years, c) self-reported severity of current symptoms on a clinical level as indicated by Beck Depression Inventory-II [[17](#_ENREF_17)] scores above 19, d) age 25 to 60 thus excluding cases of late-onset depression, and e) fluency in spoken and written German. Exclusion criteria were a) history of psychosis or mania, current eating disorder, obsessive compulsive disorder, current self-harm, current substance abuse or dependence, b) history of traumatic brain injury and c) current treatment with cognitive behavioral therapy. We allowed patients who were currently taking antidepressants into the study provided that the medication had not been changed during the last four weeks before entry into the study.

In order to be included in the healthy control group, participants had to be free of current psychiatric disorders as assessed by Structured Clinical Interview for DSM IV and had to have a BDI-II score below the threshold for minimal symptoms, i.e., BDI-II < 13.

Recruitment procedure

*N = 602* potential participants contacted the research team in response to advertisements, and were screened in a telephone interview. *N* = 85 were invited for initial assessments during which the Structured Clinical Interview for DSM-IV was conducted. *N* = 74 were found to be eligible for participation, and invited to take part in the intervention stage of the study. Depressed participants were randomly allocated to either mindfulness training or the stress-reduction training. Of the *n* = 38 participants who received the mindfulness training, *n* = 2 dropped out of the training, leaving a sample of *n* = 36. Of the *n* = 36 participants allocated to the stress-reduction condition, *n* = 4 dropped out of treatment leaving a sample of *n* = 32.

**S2: Results in the beta frequency range**

**
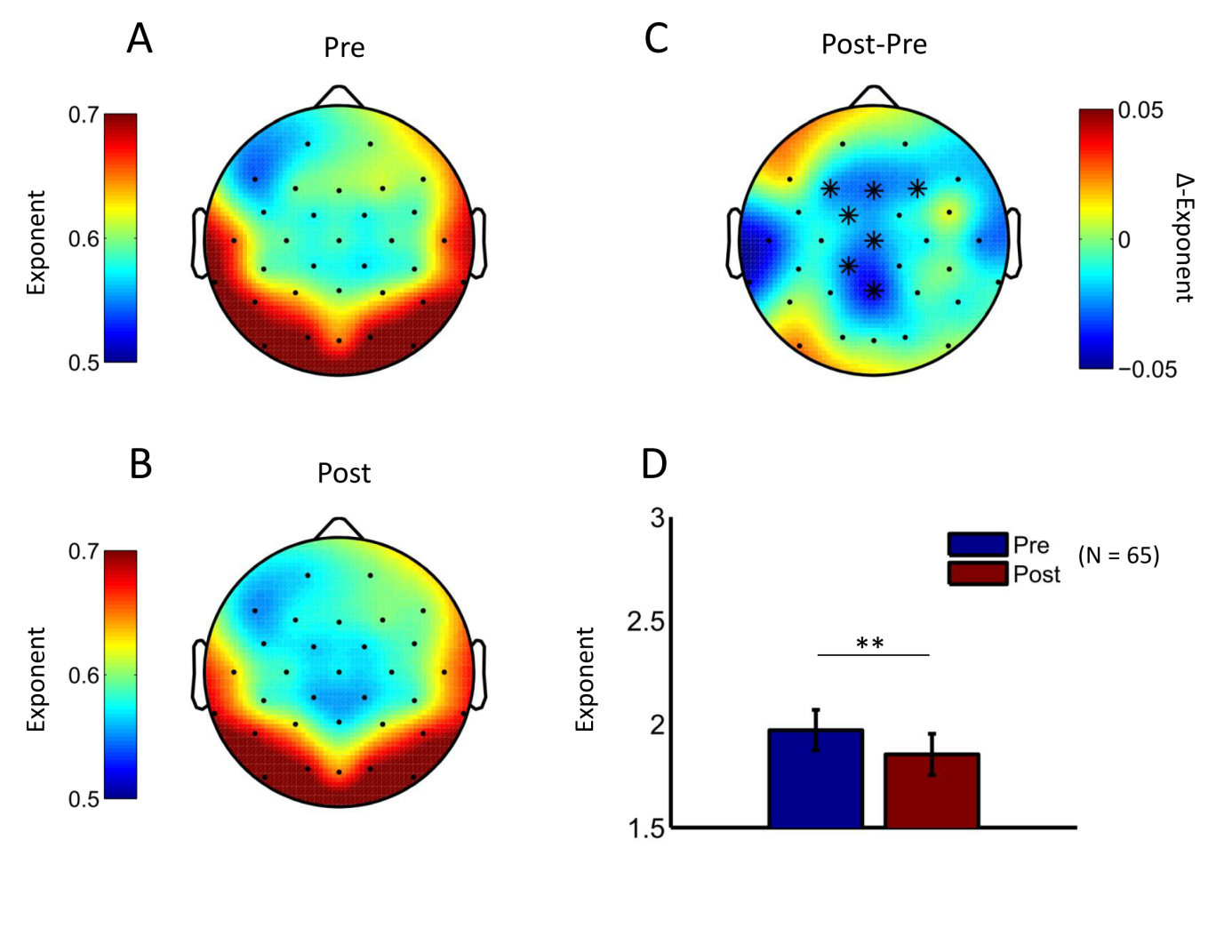
**

**Figure S2: Amplitude of beta oscillations is reduced by psychological treatment.**

Topographic distributions of the beta oscillation amplitudes at baseline (A), post-treatment (B) and their difference (C). Electrodes that were significant in the cluster statistic are marked with a star (cluster statistic, *p* = 0.016). D: Mean exponent values at the electrode of maximal difference (Pz, t-statistic, ** p < 0.01).
